# The effects of surface albedo and initial lignin concentration on photodegradation of two varieties of *Sorghum bicolor* litter

**DOI:** 10.1038/s41598-019-55272-x

**Published:** 2019-12-10

**Authors:** Christopher T. Ruhland, Joshua A. Niere

**Affiliations:** Department of Biological Sciences, TS-242 Trafton Sciences Center, Minnesota State University Mankato, Mankato, MN 56001 USA

**Keywords:** Carbon cycle, Ecophysiology, Carbon cycle

## Abstract

Decomposition of plant litter exposed to solar radiation appears to be a significant contributor to carbon cycling in some ecosystems. One factor that may influence incident solar radiation exposure on litter is surface albedo. Snow and soils with high reflectivity may enhance photodecomposition, especially in litter that stands upright for extended periods. We examined the influence of different surface albedos on the photodegradation of two varieties of sorghum (*Sorghum bicolor*) litter for 200-d, in southern Minnesota using litterbags made of material with a high transmittance of ultraviolet radiation (UV; 280–400 nm). One of these cultivars was a brown-midrib double mutant (DM) which had reduced levels of lignin compared to the wild type (WT). After 200-d sorghum litter had lost > 50% of its initial mass, and litter that was exposed to a high UV/high visible surface albedo had lost 1.4 and 2.5% more mass than litter exposed to a low UV/high visible and low UV/low visible surface albedo, respectively. Mass loss patterns agreed with initial litter chemistry, as DM litter had higher initial N, neutral detergent fiber (NDF) solubles and holocellulose:lignin ratios and lower lignin:N ratios than WT litter. Mass loss appears to be related to increased loss of hemicellulose and NDF soluble concentrations and not to lignin concentrations. Our results demonstrate that surface albedo has a small but significant effect on photodecomposition of sorghum litter.

## Introduction

The terrestrial biosphere is a major sink of carbon holding approximately 2,000 Gt of carbon, with around 1,200 Gt of this carbon held within dead biomass^1^. Decomposition of this material, a natural process, is a major contributor of carbon to the atmosphere and is an order of magnitude higher than that of fossil fuel combustion^2^. Decomposition of plant litter is governed by a hierarchy of abiotic and biotic factors such as climate, litter chemistry, and soil organisms and is easily modeled in most terrestrial systems^3^. However, these traditional paradigms do not always hold true^4–6^, and in some systems additional factors may be at play. For example, it was once assumed that decay of plant litter and subsequent carbon efflux from soil was low under freezing conditions; however new evidence suggests that activity of cold-tolerant microorganisms are important drivers for decomposition in many cold ecosystems^7–9^. In contrast, in arid and semiarid climates, decay rates of plant litter are higher than empirical models predict, and photodegradation by ultraviolet and short-wavelength visible radiation has been suggested as an additional driver for decomposition^10–13^.

Photodegradation in decomposition studies has been defined as the breakdown of organic material after exposure to solar radiation^11^, which can be facilitated through biotic and abiotic means^13,14^. Solar ultraviolet-B (UV-B; 280–320 nm), ultraviolet-A (UV-A; 320–400 nm), and short-wavelength visible (400–500 nm) wavebands all appear to play a role in photodegradation and may facilitate the breakdown of organic material through several different mechanisms (reviewed in King *et al*.^11^). In a recent meta-analysis of photodegradation studies, King *et al*.^11^ estimates that exposure to solar radiation increases mass loss of plant litter by 23%. The primary target of solar radiation appears to be lignin, which has a strong absorption coefficient in the UV-B portion of the spectrum^15,16^. However several studies have also found increased loss of cellulose^17^, hemicellulose^13^, and water-soluble fractions^13^ in litter associated with UV-B exposure. Whether this breakdown is mediated through direct photolysis or mediated through a reactive intermediate (“indirect photolysis”) is still unknown.

In the same meta-analysis King *et al*.^11^ also highlights several factors that may affect solar radiation exposure and subsequent photodecomposition of plant litter. One factor that appears to have received little attention is the influence of surface albedo. For example, Rozema *et al*.^18^ suggested that soils with high surface reflectivity, such as sandy soils, may enhance photodegradation rates of litter placed adjacent to them. King *et al*.^11^ also speculated that other surfaces with high reflectance, such as snow, might accelerate decomposition of plant litter. In this study, we examine the influence of different surface albedos on photodecomposition of two different cultivars of *Sorghum bicolor* litter (wild type versus a low-lignin mutant) in Southern Minnesota over a 200-d period. We used litter from the brown midrib mutation (bmr) of sorghum which has impaired activity of cinnamyl alcohol dehydrogenase (CAD) and/or caffeate/5-hydroxyferulate-*O*-methyl transferase (COMT) enzymes^9,19,20^ resulting in reduced and/or altered amounts of *p*-hydroxyphenyl (H), guaiacyl (G), and syringyl (S) subunits of lignin compared to wild-type plants. We suspected that litter deriving from wild-type plants that have initially higher lignin content would have increased mass-loss rates and more alterations in cell-wall chemistry than the low-lignin bmr litter as a result of direct photolysis of lignin.

## Results

### Initial Litter Chemistry

There were several differences in initial litter quality between the two *Sorghum* cultivars (Table 1). Initial cellulose and lignin concentrations were 4.4 and 28.8% higher in the WT than in DM, respectively, while NDF solubles were 3.7% lower (Table 1; *t*-test*; P* < 0.05). Initial concentrations of hemicellulose averaged 23.1% and were similar between the two cultivars. Interestingly, initial N concentrations were 26% higher in the DM cultivar. Compared to the WT, litter from DM had higher ratios of holocellulose (cellulose + hemicellulose):lignin and lower C:N and lignin:N (Table 1; *t*-test; *P* < 0.05).

**Table 1:** Initial chemistry of wild type (WT) and the stacked double mutant (DM; *bmr6/12*) varieties of *Sorghum bicolor* litter.

Parameter	WT	DM	P
Carbon (%)	38.48 (0.78)	38.55 (0.46)	0.310
Nitrogen (%)	0.96 (0.05)	1.23 (0.07)	<0.001
C:N	40.73 (1.84)	32.14 (1.60)	<0.001
NDF Solubles (%)	43.49 (0.35)	45.14 (0.64)	0.036
Hemicellulose (%)	22.81 (0.19)	23.39 (0.25)	0.444
Cellulose (%)	31.63 (0.27)	30.25 (0.52)	0.003
Lignin (%)	3.33 (0.11)	2.37 (0.20)	0.003
Lignin:N	2.91 (0.14)	1.49 (0.08)	<0.001
Holocellulose:Lignin	18.54 (1.28)	27.71 (3.03)	0.012

Values are means (± SE; n = 9 or 10). P-values were calculated using a two-tailed t-test.

### Microclimate and mass loss

**Figure 1:**
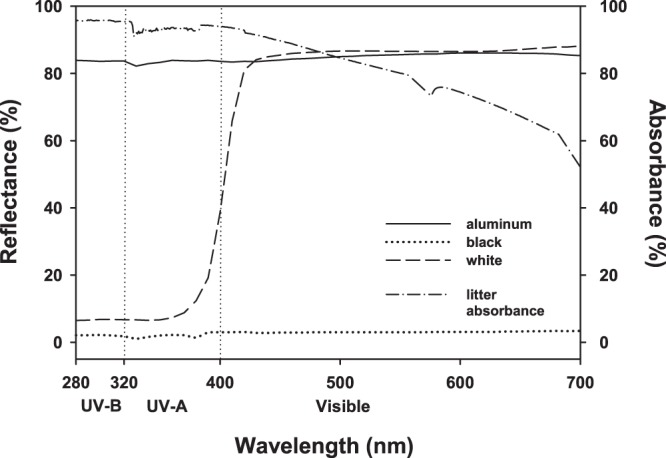
Spectral reflectance from 280 to 700 nm of three surfaces representing high UV/high visible albedo (“aluminum”), low UV/high visible albedo (“white”), and low UV/low visible albedo (“black”) and representative average spectral absorbance of sorghum litter (adaxial + abaxial). Measurements were made using a 50-mm integrating sphere (Labsphere RSA-PE-20) and compared against a NIST-traceable standard (Labsphere USRS-99-010). Litter absorbance (%) = 100 - %transmittance - %reflectance.

Temperatures of litter inside litterbags were recorded for 30-d and averaged 1.8 °C warmer than the measured ambient air temperature. Average temperatures, over a 24-hour period, were not different between the three surfaces (Kruskal Wallis, *P* > 0.05; *data not shown*), even though black surfaces had a lower average surface albedo (Fig. 1). Minimum daily temperatures were not different between the three surfaces; however maximum daily temperatures inside litterbags over black surfaces was between 0.8–3.2 °C higher than in litterbags over aluminum and white surfaces (Kruskal Wallis, *P* < 0.05; *data not shown*). Average, minimum, and maximum humidity was not different between treatments and averaged 56.6, 36.4 and 76.2%, respectively (*data not shown*).

**Figure 2:**
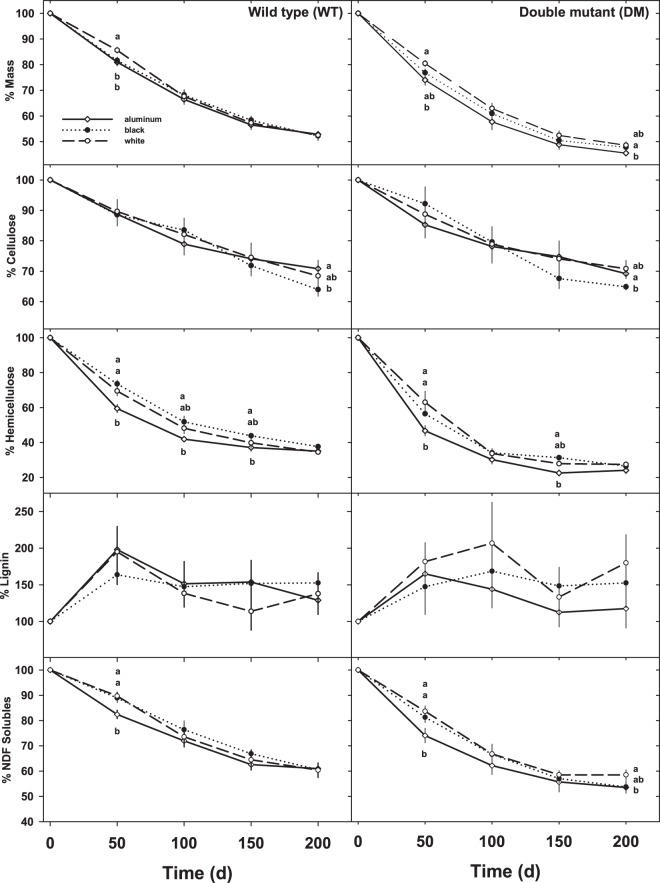
The percent dry mass, cellulose, hemicellulose, lignin, and NDF solubles remaining of wild type (WT) and a *bmr6*/*bmr12* stacked hybrid (double mutant; DM) sorghum cultivars over the course of the experiment. See Fig. 1 for treatment explanations. Values are means (+ /− 1SE; n = 6). Different letters designate a significant difference between treatments on a specific sampling date (*P* < 0.05).

**Figure 3:**
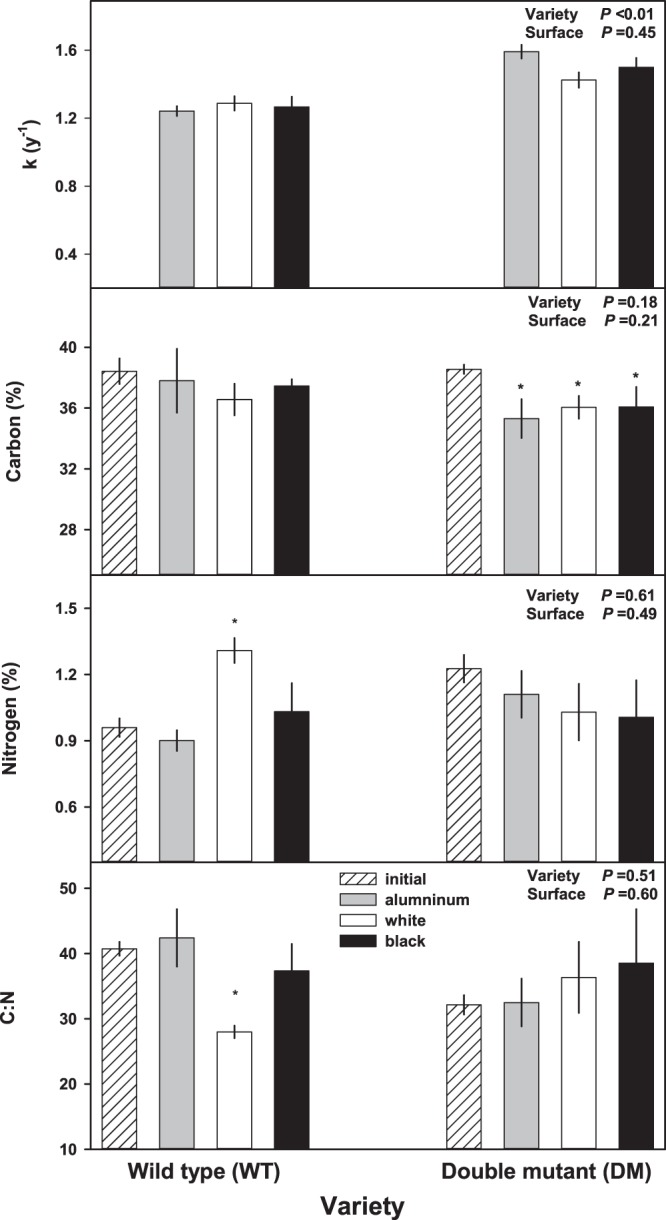
Exponential decay rates (k), % carbon, %nitrogen, and C:N of WT and DM sorghum litter cultivars after 200 days. See Fig. 1 for treatment explanations. Values are means (+ /− 1SE; n = 6 for k and n = 3 for %C, %N, and C:N). An * designates differences between initial and final values (*P* < 0.05).

Surface treatment, variety, and time all had significant effects on mass loss (ANOVA, *P* < 0.01; Table 2). In addition, there was also a significant time*variety interaction on mass loss (Table 2). Across both varieties, sorghum litter on aluminum surfaces on average lost 1.4 and 2.5% more mass than those over black and white surfaces, respectively (LSD, *P* < 0.05; Fig. 2). In addition, DM litter lost 5% more mass than did the WT after 200-d, with WT losing an average of 47.5% of initial mass and DM losing an average of 52.6% across all surface types (LSD, *P* < 0.05; Fig. 2). After 50-d, WT litter on the aluminum and black surfaces lost 4–5% more mass than that on the white surfaces (Kruskal Wallis, *P* < 0.05; Fig. 2). The DM litter on the aluminum surface lost 6.5% more mass than the white surface after 50-d and 3.1% more than the black surface after 200 d (Kruskal Wallis, *P* < 0.05; Fig. 2). There were no other effects of surface reflectance on mass loss. Patterns of mass loss were exponential for both varieties (Fig. 2), and r^2^ values ranged from 0.84 to 0.99 for decay models (*data not shown*). Not surprisingly, decay constants were 15% higher in DM litter than in WT (LSD, *P* < 0.05; Fig. 3).

**Table 2:** ANOVA for variety, surface albedo, and time effects on measured parameters in the wild type (WT) and *bmr6/bmr12* double mutant (DM) varieties of *Sorghum bicolor* after 200 days.

Parameter	Treatment effect	df	F	P
Mass remaining (%)	**Time**	4	1117.5	**<0.01**
	**Variety**	1	79.9	**<0.01**
	**Surface**	2	6.7	**<0.01**
	**Time** × **Variety**	4	5.4	**<0.01**
	Time × Surface	8	1.1	0.35
	Variety × Surface	2	1.6	0.19
	Time × Variety × Surface	8	0.3	0.98
Cellulose remaining (%)	**Time**	4	81.8	**<0.01**
	Variety	1	0.3	0.56
	Surface	2	0.5	0.63
	Time × Variety	4	0.2	0.94
	Time × Surface	8	0.8	0.59
	Variety × Surface	2	0.1	0.96
	Time × Variety × Surface	8	0.3	0.97
Hemicellulose remaining (%)	**Time**	4	1061.6	**<0.01**
	**Variety**	1	157.2	**<0.01**
	**Surface**	2	20.3	**<0.01**
	**Time** × **Variety**	4	10.9	**<0.01**
	**Time** × **Surface**	8	3.7	**<0.01**
	Variety × Surface	2	1.9	0.15
	Time × Variety × Surface	8	0.7	0.65
Lignin remaining (%)	**Time**	4	7.3	**<0.01**
	Variety	1	0.1	0.96
	Surface	2	0.5	0.61
	Time × Variety	4	0.8	0.55
	Time × Surface	8	0.6	0.75
	Variety × Surface	2	1.7	0.19
	Time × Variety × Surface	8	0.2	0.99
NDF solubles remaining (%)	**Time**	4	398.1	**<0.01**
	**Variety**	1	55.3	**<0.01**
	**Surface**	2	6.7	**<0.01**
	**Time** × **Variety**	4	3.9	**<0.01**
	Time × Surface	8	1.5	0.15
	Variety × Surface	2	1.2	0.31
	Time × Variety × Surface	8	0.2	0.99

### Litter Constituents

Cellulose fractions declined steadily over the course of the experiment, and after 200 d approximately 29–36% of cellulose was lost in both varieties (Fig. 2). At the final census, cellulose fractions of litter suspended over aluminum surfaces averaged 70.1 and 69.2% of initial concentrations for WT and DM, respectively, compared to 64.0 and 64.9% of initial values in litter suspended over black surfaces (Kruskal Wallis; *P* < 0.05; Fig. 2). Hemicellulose concentrations declined rapidly over the course of the experiment, and after 200-d approximately 63–76% of the initial hemicelluloses were lost (Fig. 2).

There were significant variety and surface treatment effects on hemicellulose concentrations (ANOVA; P < 0.01; Table 2). Hemicellulose losses were most rapid within the first 50 d, and sorghum litter suspended over aluminum surfaces had lost 10–17% more of the initial hemicellulose than litter suspended over black and white surfaces for both varieties (Kruskal Wallis; *P* < 0.05). Concentrations of lignin were inconsistent throughout the experiment as concentrations increased (exceeded 100% of initial values) after the first harvest date (Fig. 2). Not surprisingly, fractions of NDF solubles decreased over the course of the experiment (Fig. 2), and there were significant variety and surface treatment effects on the amount of NDF solubles remaining (ANOVA; *P* < 0.05; Table 2). Litter from DM lost more soluble compounds than WT (LSD; *P* < 0.05), and litter from aluminum surfaces had lower concentrations than black and white surfaces in both varieties (LSD; *P* < 0.05). There were no significant variety or surface treatment effects on final concentrations of C, N or C:N (Fig. 3).

## Discussion

Surface albedo had a small, but significant, effect on final mass loss on sorghum litter (Table 2). The high reflectivity of both UV and visible radiation of aluminum surfaces (Fig. 1) resulted in an average mass loss that was 1.4 and 2.5% greater than of those over white (low UV/high visible albedo) and black (low UV/ low visible albedo) surfaces, respectively. These effects were more pronounced in the low-lignin DM where litter over aluminum surfaces lost 2.3 and 3.1% more mass, respectively, than those under black or white surfaces at the end of the experiment (Fig. 2). Previous studies have found that UV-B^10,17,21^, UV-A^22^ and lower-wavelength visible^13,16^ radiation all play a role in the breakdown of plant litter; however the mechanism behind this photodegradation remains unknown. Lignin has been proposed to be a primary target for photodegradation of plant litter^18^. Lignin is a complex polymer consisting of aromatic phenolic subunits that are products of the phenylpropanoid pathway. These phenolic subunits all have strong absorption in the UV-B and UV-A wavebands^23^, and lignocellulosic mixtures have appreciable absorption in the visible portion of the electromagnetic spectrum^16^. However mass loss in our experiment was 5% greater in DM than WT (Fig. 2; Table 2) despite having lower lignin concentrations (Table 1) suggesting that direct photolysis of lignin is not a major driver of photodecomposition in sorghum litter. The increased mass loss in the DM over WT is consistent with presumed initial litter chemistry^24^ and may be more related to the higher initial N, or ratios of lignin:N, holocellulose:lignin or C:N between the two sorghum varieties^9^ (Table 1). King *et al*.^11^ noted that the initial lignin content and the magnitude of photodegradation are not necessarily coupled, suggesting that other poorly understood mechanisms are responsible for UV-induced mass loss of plant litter. Additionally, concentrations of lignin increased from initial values over the course of the experiment (Fig. 2), and previous studies have suggested this increase may be the result of a buildup of acid-insoluble byproducts through the decomposition process. Therefore experiments examining changes in lignin concentrations over time should be interpreted cautiously^25–27^.

The effect of surface albedo also influenced hemicellulose concentrations in both varieties of sorghum litter (Table 2). Litter suspended over the aluminum surfaces had 4.7% and 5.8% lower hemicellulose concentrations on average than those over white and black surface treatments, respectively (Fig. 2). Results from other studies have demonstrated considerable loss of hemicelluloses in litter exposed to UV^13^. These hemicelluloses are responsible for binding cellulose fibrils together in the cell wall matrix, and the accelerated loss of these hemicelluloses relative to other constituents may accelerate the photodegradation process. Concentrations of hemicellulose were correlated with mass loss over the course of the experiment (r^2^ = 0.61; *data not shown*), and on average the DM lost 5.8% more than WT (Fig. 2), even though initial concentrations were similar (Table 1). In a recent study, Day *et al*.^13^ suggests that initial concentrations of hemicellulose may be an important predictor in the sensitivity of plant litter to photodegradation although the mechanism behind the breakdown of this constituent remains unknown.

In an experiment that examined photodegradation of plant litter in the Sonoran Desert, Day *et al*.^13^ noted that exposure to sunlight increased the amount of water-soluble materials leaching from litter, and this effect increased over the course of 3 years. In addition, they noted that microbial respiration rates were positively correlated to these water-soluble fractions (but not NDF solubles) under full sunlight, which could potentially explain the accelerated mass loss in these treatments. While we did not examine warm-water extractable solubles in our experiment, we did observe that litter on aluminum surfaces lost 2.8–3.2% more of their initial NDF solubles than those over black or white surfaces in both sorghum varieties (LSD, *P* < 0.05; Table 2). The NDF technique we employed removes proteins, sugars, lipids, and pectins^28^ and would undoubtedly remove more cellular materials than warm-water extraction alone. Interestingly, in our experiment concentrations of NDF solubles were highly correlated with mass loss (r^2^ = 0.87; *data not shown*), and the leaching of these labile compounds could accelerate decomposition.

Our surface treatments provided the opportunity to examine different combinations of UV- and visible-radiation albedo effects on the process of photodegradation of sorghum litter. For example, our aluminum-surface treatments mimic the high reflectivity of snow^29,30^ in both the UV and visible wavebands of solar radiation, and sorghum litter suspended over these treatments lost more mass than other treatments (Fig. 1). Stover and other crop residues are often left to overwinter on soils covered in snow in Minnesota, and exposure to enhanced levels of solar radiation due to increased albedo may prime litter for subsequent microbial breakdown in the early spring. While we did not measure indirect radiation intercepted by litter in this experiment, we suspect that increased exposure to UV and/or visible radiation above the aluminum treatments enhanced mass loss. Lastly, while our experiment was relatively short (200 d), it appears that surface albedo might be an additional factor to consider when modeling photodecomposition of plant litter; especially in systems where surface albedo is high for longer periods of time.

In conclusion, our study shows that surface albedo of UV has a significant effect on photodegradation of sorghum litter. This photodecomposition does not appear to be related to the initial lignin concentration of litter between the WT and DM cultivars, suggesting that direct photolysis of lignin is not a major driver for mass loss in sorghum. However, mass loss was correlated with concentrations of hemicellulose and NDF solubles. Future studies should examine how the UV-induced loss of hemicelluloses influences the breakdown of the cell wall lignocellulosic matrix and subsequent mass loss of sorghum litter.

## Methods and Materials

### Litter collection and litterbag design

Seeds of wild type (WT), *bmr6* (impaired CAD activity), and *bmr12* (impaired COMT activity) cultivars of sorghum were obtained from the USDA-ARS at the University of Nebraska, Lincoln^31^, and a stacked *bmr6/bmr12* hybrid (“double mutant;” DM) was created following the methods of Pedersen *et al*.^32^ as described in Ruhland *et al*.^9^. Seeds were planted in 0.19-m^3^ pots on 1 July 2014 and plants were grown in a greenhouse at Minnesota State University (44°08′N; 93°60′W) until death. Plants were air dried for >30 d and aboveground biomass was harvested and separated into leaves, stems, and reproductive structures. Leaves were cut into pieces 15 cm long, placed in paper bags, and oven dried at 60 °C for >48 h.

Two grams of litter were placed into 46 × 18 cm Aclar envelopes (Aclar Type 22 A film, Proplastics, Linden, NJ, USA) following Day *et al*.^10^. Aclar was chosen due to its ability to transmit 87–89% of UV-B (280–315 nm), 89–92% of UV-A (315–400 nm), and 92–93% of photosynthetically active radiation^33^ (PAR, 400–700 nm). One hundred small holes (1-mm diameter) were added to each envelope to allow air and water to reach litter. Litterbags were placed upon each surface on the roof of the Trafton Science Center at Minnesota State University on 12 June 2016. Eight litterbags (four WT and four DM) were randomly placed 1.27 cm above each treatment surface (see below).

### Treatment surfaces and collection dates

The surface albedo of soil (dark, organically rich) is approximately 2%, coarse sand (0.2–2.0 mm) is approximately 9% and snow is between 74–94% depending on age and moisture^29,30^. Using a UV/visible spectrometer (Lambda 35, Perkin Elmer Incorporated, Waltham, MA, USA), equipped with a 50-mm machined integrating sphere (RSA-PE-20, Labsphere Incorporated, North Sutton, NH USA), reflectance of several artificial surfaces was measured in order to determine surface covers that best mimicked these natural surfaces. Measurements were taken between 280–760-nm and were compared against a NIST-traceable standard (USRS-99–010, Labsphere Incorporated). It was determined that the artificial covers that best mimicked the natural surfaces, when applied to a plywood base, were 0.024-mm thick aluminum foil (Reynold’s Wrap, Lake Forest IL, USA), flat black paint (exterior flat black, Glidden, Strongsville OH, USA), and flat white paint (exterior flat white, Glidden, Strongsville OH, USA). The surface covered in aluminum-foil (“aluminum”) reflected between 84–85% of visible, UV-A, and UV-B (high UV/high visible albedo) radiation and was similar to freshly fallen dry snow. The white-painted surface (“white”) reflected ≈86% of visible and only ≈6% of both UV-A and UV-B (low UV/ high visible albedo) and was similar to dry coarse sand. The black-painted surface (“black”) only reflected between 2–3% of visible, UV-A, and UV-B (low UV/low visible albedo; Fig. 1) and was similar to a dark soil.

Eighteen surfaces were constructed out of plywood (1.2 m × 1.2 m) with legs that elevated the surfaces 10.2-cm above the ground in order to allow airflow under the surfaces. Each of the three artificial surface types were applied to six plywood surfaces. Three coats of each paint type were applied, and the aluminum foil was attached to the plywood using staples. Litterbags were suspended above each surface using wooden dowels that were placed on each treatment surface. Litterbags were placed perpendicular to treatment surfaces in order to mimic litter stover that remains in the field following sorghum harvest. Treatment plots were placed in a SE direction on the roof of Trafton Science Center (44°08′N; 93°60′W). During the experiment, litter temperatures and relative humidity inside of bags were recorded within litterbags and on the surfaces using a data logger (U23 Pro V2, Onset HOBO, Boume, MA, USA) with a 0.5-cm external temperature sensor. Temperature measurements were taken every 5 min and averaged each hour over the month of August 2016, and humidity measurements were made every minute and averaged each hour from 6 March to 13 March 2017.

Thirty-six litterbags (eighteen per variety, one of each variety per surface) were collected at 50, 100, 150, and 200 days (31 July, 19 September, 8 November, and 28 December 2016) after the start of the experiment. Following collection, the litter was removed from the litterbags and oven-dried at 60 °C for >48 h prior to being weighed for mass loss and subsequent chemical analyses (see below).

### Litter chemistry

On each collection date, concentrations of hemicellulose, cellulose, and lignin were determined using a sequential extraction technique^28^ in a fiber analyzer (model A200; ANKOM Technology, Macedon NY, USA) following Warnke and Ruhland^34^. Samples were ground with a Wiley Mill (1-mm mesh screen), and approximately 0.50 g (±0.05 g) of ground litter was placed into filter bags (F57; ANKOM Technology, Macedon NY, USA). Litter samples were digested sequentially in a neutral detergent fiber solution (NDF) to estimate concentrations of cellulose + hemicellulose + lignin and then in an acid detergent fiber solution (ADF) to determine concentrations of cellulose + lignin. Lastly, samples were agitated in 72% (v/v) H_2_SO_4_ for 3 h and then placed in a muffle furnace (600 °C) to determine lignin (ADL) and ash concentrations. Cellulose concentrations were calculated as %ADF - %ADL, and hemicellulose concentrations were calculated as %NDF - %ADF. A more detailed description of this technique can be found in Warnke and Ruhland^35^. Additionally, we estimated concentrations of C and N in initial and final litter samples using a flash combustion analyzer (Tru Spec, Leco Corporation, St. Joseph, MO, USA).

### Statistical Analyses

For mass loss and litter chemistry analyses, a repeated measures Analysis of Variance (ANOVA) was used that took into account variety, surface treatments, and census date followed by a Kruskal-Wallis or Least-Squared Difference (LSD) test. A two-way ANOVA was used to examine differences in C, N, C:N, and decay constants (k) between varieties and surface treatments at the end of the experiment. A Student’s *t*-test (SigmaPlot 13, 2015) was used in order to measure initial differences in litter chemistry between cultivars. Decay constants (k) were calculated using a single exponential decay model where X_t_/X_0_ = e^−kt^, where X_0_ and X_t_ are the initial and sample mass at time t following Day *et al*.^35^. Decay constants were calculated for each treatment surface (n = 6) using SigmaPlot. Differences were considered significant at the *P* *<* 0.05 level.

## Data Availability

The datasets collected, graphed, and analyzed are available from the corresponding author upon request.
